# L‐Arginine Increases Postprandial Circulating GLP‐1 and PYY Levels in Humans

**DOI:** 10.1002/oby.22323

**Published:** 2018-10-25

**Authors:** Anjali Amin, Christina Neophytou, Shermaine Thein, Niamh M Martin, Amin Alamshah, Eleanor Spreckley, Stephen R. Bloom, Kevin G. Murphy

**Affiliations:** ^1^ Section of Endocrinology and Investigative Medicine, Department of Medicine Imperial College London London UK

## Abstract

**Objective:**

The satiating effect of protein compared with other nutrients has been well described and is thought to be mediated, in part, by gut hormone release. Previously, it has been shown that oral L‐arginine acts as a GLP‐1 secretagogue both *in vitro* and *in vivo* in rodents. Here, the effect of L‐arginine on gut hormone release in humans was investigated.

**Methods:**

The hypothesis was tested in two separate studies. The first study assessed the tolerability of oral L‐arginine in healthy human subjects. The second study assessed the effect of oral L‐arginine on gut hormone release following an *ad libitum *meal. Subjects were given L‐arginine, glycine (control amino acid), or vehicle control in a randomized double‐blind fashion.

**Results:**

At a dose of 17.1 mmol, L‐arginine was well tolerated and stimulated the release of plasma GLP‐1 (*P* < 0.05) and PYY (*P* < 0.001) following an *ad libitum* meal. Food diaries showed a trend toward lower energy intake and particularly fat intake following L‐arginine treatment.

**Conclusions:**

L‐arginine can significantly elevate GLP‐1 and PYY in healthy human volunteers in combination with a meal. Further work is required to investigate whether L‐arginine may have utility in the suppression of appetite and food intake.

## Introduction

The satiating effect of protein is greater than that of other macronutrients 1, 2, 3, 4. High‐protein diets reduce food intake, facilitate weight loss, and improve body composition in animal models and humans 5, 6, 7, 8. These effects have been suggested to be mediated by processes including the modulation of energy expenditure 9 and hepatic gluconeogenesis 10, but the precise mechanisms involved remain unclear. There is evidence to suggest that protein influences gastrointestinal hormones to alter satiety. Protein has been reported to increase levels of specific anorectic gut hormones to a greater extent than other macronutrients 11, 12. Peptide YY (PYY) and glucagon‐like peptide‐1 (GLP‐1) are released from endocrine cells in the gut in response to food intake and reduce food intake following peripheral administration in animals and humans 13, 14. A high‐protein meal results in greater increases in circulating concentrations of both PYY and GLP‐1 in humans with normal weight when compared with isocaloric high‐fat or high‐carbohydrate meals 15. Mice lacking PYY are resistant to the body‐weight‐reducing effect of a high‐protein diet 12.

Specific nutrients are detected within the gut and by peripheral nerves to inhibit food intake in both humans and animal models 16, 17. Cells in the gut epithelial lining, which have direct contact with the intraluminal contents and include enterocytes, brush cells, and enteroendocrine cells, have chemosensory properties. Enteroendocrine cells play a specialized role in luminal nutrient sensing, although they represent less than 1% of epithelial cells within the gut. Peptide hormones are released from secretory granules located in the basal cytoplasm of this cell type 18. Specifically, following a meal, enteroendocrine L‐cells secrete the peptide hormones GLP‐1 and PYY_1‐36_, which is subsequently processed into the form PYY_3‐36_. Both GLP‐1 and PYY_3‐36_ reduce food intake and are involved in the regulation of energy homeostasis 19.

Evidence has suggested that the amino acid products of protein digestion may be sensed both peripherally within the gut and centrally to regulate energy intake 20. Individual amino acids have been shown to influence gastrointestinal hormone release and appetite 21, 22. G‐protein coupled receptor family class C amino‐acid‐sensing receptors are present in the gastrointestinal tract, where some are known to be expressed on enteroendocrine L cells. These receptors, which include the calcium‐sensing receptor, the T1R1/T1R3 heterodimeric receptor, and the GPRC6A receptor, are known to promiscuously bind several L‐amino acid ligands and thus appear suited to detect the varied products of protein digestion. Supplementing foods with specific amino acids selected for their appetite‐reducing effect may represent a novel approach for the prevention of weight gain or the treatment of obesity. The eventual goal would be to design foods or dietary regimens that cause an increased sense of fullness and encourage the individual to stop eating sooner, thus reducing total energy intake 23, 24. Our aim in this study was to investigate the effects of an amino acid administered at physiological levels (i.e., similar to the amount present in a high‐protein meal) on anorectic gut hormone release and the regulation of appetite.

L‐arginine appeared a good candidate for an appetite‐reducing amino acid. L‐arginine is a conditionally essential amino acid that can activate all three of the known promiscuous amino‐acid‐sensing receptors and, in particular, the T1R1/T1R3 receptor in rodents 25. Previous work has shown specific amino acids can influence food intake and gut hormone release. For example, glutamine stimulates GLP‐1 secretion from an enteroendocrine cell line 26, and studies have suggested that L‐glutamine stimulates the release of GLP‐1 in humans 22, 27, 28. The amino acid L‐arginine also reduces food intake and elevates circulating levels of GLP‐1 and PYY in rodents 29, 30, 31, while other L‐amino acids, including glycine, have no effect 21. There is evidence that oral L‐arginine acts as a GLP‐1 secretagogue both *in vitro* 32 and *in vivo* in rodents 30. However, to date, there has been no evidence of this effect in humans. We investigated the effect of L‐arginine on circulating levels of appetite‐modulating gastrointestinal hormones in humans and the consequent effect on appetite.

## Methods

### Study participants

Studies were conducted following ethical approval (West London Research Ethics Committee 1, London, UK) and according to the principles of the Declaration of Helsinki. All participants gave written informed consent prior to study enrollment.

### Pilot study on tolerability of L‐arginine (study 1)

Healthy male (*n* = 1) and female (*n* = 6) subjects with a mean age of 39.4 (SD 11.4) years and BMI of 24.6 (SD 4.7) kg/m^2^ who had been weight stable for 3 months prior to study enrollment were recruited (Table 1.

**Table 1:** Baseline characteristics for participants in the pilot study on the effect of L‐arginine on gut hormone release and tolerability

	**All subjects**
**Age (y)**	39.4 ± 11.4
**Female:Male**	6:1
**Weight (kg)**	68.9 ± 17.3
**Height (m)**	1.67 ± 0.08
**BMI (kg/m^2^)**	24.6 ± 4.7
**Blood pressure (mm Hg)**	112/68 ± 13/8

Data represent mean ± SD.

Subjects attended four study visits each separated by at least 1 week. The first visit was an acclimatization visit, the results of which were not included in the final analysis. The evening before each study visit, subjects were requested to consume an identical meal, supplied by the experimenters, at approximately 8 pm. Subjects then reported to the clinical research facility at 8:30 am the following day, having fasted from 9 pm the night before. On arrival, subjects were cannulated in the antecubital fossa for serial blood sampling and asked to consume hypromellose capsules containing a total of 17.1 mmol of L‐arginine hydrochloride or 17.1 mmol of glycine (Euro‐OTC‐Pharma, Horsham, UK) or empty capsules (vehicle) in a double‐blind randomized order at time (t) = 0 minutes. Glycine was chosen as a control amino acid, as this amino acid has been shown to have no effect on food intake in rodent studies 21. This dose of L‐arginine was chosen as it was similar to levels of L‐arginine found in a high‐protein meal. Hypromellose capsules, which are relatively inert and break down in the stomach, were used to deliver the amino acids 33. In addition to tolerability, we carried out a pilot analysis of gut hormone release and subjective appetite. Blood samples were taken at 15‐minute intervals commencing at t = −15 minutes for 2.5 hours after dosing for the measurement of plasma acylated ghrelin, GLP‐1, and PYY. Subjects were asked to complete visual analogue scales (VAS) at the corresponding time points. Participants rated subjective feelings of hunger (“How hungry do you feel right now?”), pleasantness to eat (“How pleasant would it be to eat right now?”), prospective food intake (“How much could you eat right now?”), fullness (“How full do you feel right now?”), and sickness (“How sick do you feel right now?”) using a 100‐mm horizontal VAS at −15, 0, 15, 30, 45, 60, 75, 90, 105, 120,135, and 150 minutes following ingestion of L‐arginine. Subjects were also asked to report any additional side effects that occurred during the visit or after the study visit was completed.

### Effect of L‐arginine on gut hormone release and food intake following an *ad libitum* meal (study 2)

With 80% power, it was predicted that nine subjects would be required for this study based on a difference in gut hormone release of 30%. This is the approximate magnitude of increase in PYY release observed following a high‐protein meal compared with a high‐carbohydrate meal 12, 15. Healthy male (*n* = 1) and female (*n* = 8) subjects with a mean age of 36.0 (SD 10.8) years and BMI of 25.1 (SD 3.5) kg/m^2^ who had been weight stable for 3 months prior to study enrollment were recruited (Table 2.

**Table 2:** Baseline characteristics for participants in the study of the effect of L‐arginine on gut hormone release and food intake following an *ad libitum* meal

	**All subjects**
**Age (y)**	36.0 ± 10.8
**Female:Male**	8:1
**Weight (kg)**	69.4 ± 16.4
**Height (m)**	1.65 ± 0.09
**BMI (kg/m^2^)**	25.1 ± 3.5
**Blood pressure (mm Hg)**	113/74 ± 6/5

Data represent mean ± SD.

The study design was as per study 1 (with administration of L‐arginine, glycine, or vehicle at t = 0 minutes), except that at t = 60 minutes, subjects were presented with an *ad libitum* meal, which they were asked to consume within 30 minutes. The *ad libitum* meal was served in excess, and participants were asked to eat until they were comfortably full. Participants were isolated during this part of the study. Food was weighed before and after the *ad libitum *meal, and energy intake was calculated from the manufacturer’s nutritional information. The meal given was a commercially available margherita pasta bake ready meal consisting of 1.83 kcal, 0.079 g protein, 0.196 g carbohydrate, and 0.078 g fat per gram. There were no blood samples or VAS taken at t = 75 minutes in this study. As the initial study detected no evidence of any effect on ghrelin levels, ghrelin was not measured in this second study. Subjects were asked to complete food diaries for the rest of the day and the subsequent day following their departure from the clinical research facility. Energy intake and macronutrient intake were established for each subject from analysis of completed food diaries. Food diaries were analyzed using Dietplan 6 nutritional analysis software (Forestfield Software, Horsham, UK).

### Assays

Plasma acylated ghrelin was measured using a commercially available enzyme‐linked immunosorbent assay (ELISA) kit (Merck Millipore, Burlington, Massachusetts). Plasma GLP‐1 and PYY were measured using established in‐house radioimmunoassays 34, 35. The GLP‐1 antibody has 100% cross‐reactivity with all amidated forms of GLP‐1 but does not cross‐react with glycine extended forms. The PYY antibody has 100% cross‐reactivity with PYY_1‐36_ and PYY_3‐36_. The intra‐assay coefficients of variation for GLP‐1 and PYY assays were 5.6% and 5.0%, respectively.

### Statistical analysis

Acute food intake and area under the curve (AUC) data are expressed as mean ± SEM and were analyzed by one‐way analysis of variance (ANOVA) and post hoc Bonferroni correction. Data from gut hormones and VAS were analyzed by repeated‐measures ANOVA and post hoc Bonferroni correction. The relationship between the variables under study was assessed using the Pearson coefficient of correlation *R*. *P* < 0.05 was considered significant. GraphPad Prism software (Prism 5.01; GraphPad Software Inc., San Diego, California) was used for all analyses.

## Results

### Pilot study on tolerability of L‐arginine (study 1)

#### L‐arginine was well tolerated.

There were no self‐reported side effects with the administration of oral L‐arginine. In addition, there were no significant changes on the VAS regarding nausea.

#### L‐arginine alone had no significant effect on circulating ghrelin, GLP‐1, or PYY levels.

**Figure 1:**
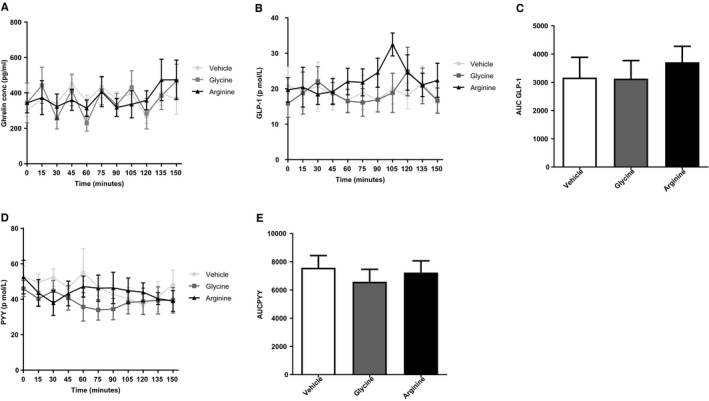
Effect of L‐arginine on gut hormone release in healthy volunteers. Results of oral ingestion of vehicle, 17.1 mmol of arginine, or 17.1 mmol of glycine (*n *= 7) on (**A**) acylated ghrelin, (**B**) AUC of acylated ghrelin, (**C**) GLP‐1, (**D**) AUC of GLP‐1, (**E**) PYY, and (**F**) AUC of PYY. Data are expressed as mean ± SEM.

L‐arginine had no significant effect on plasma ghrelin (Figure 1A‐1B), which was in accord with animal studies performed within our group (unpublished data). There was a trend for an increase in plasma GLP‐1 following ingestion of L‐arginine, though this effect did not achieve statistical significance (Figure 1C‐1D). There was no significant increase in plasma PYY following ingestion of L‐arginine (Figure 1E‐1F).

#### L‐arginine had no significant effect on subjective measures of appetite.

There was no significant difference in subjective measures of appetite using VAS following ingestion of vehicle, 17.1 mmol of arginine, or 17.1 mmol of glycine.

Following observations that L‐arginine was well tolerated with no reported side effects and that it resulted in a trend for an increase in plasma GLP‐1 release, we investigated whether L‐arginine could increase gut hormone release and influence food intake.

### Effect of L‐arginine on gut hormone release and food intake following an *ad libitum* meal (study 2)

#### L‐arginine stimulated plasma GLP‐1 and PYY following an ad libitum meal.

**Figure 2:**
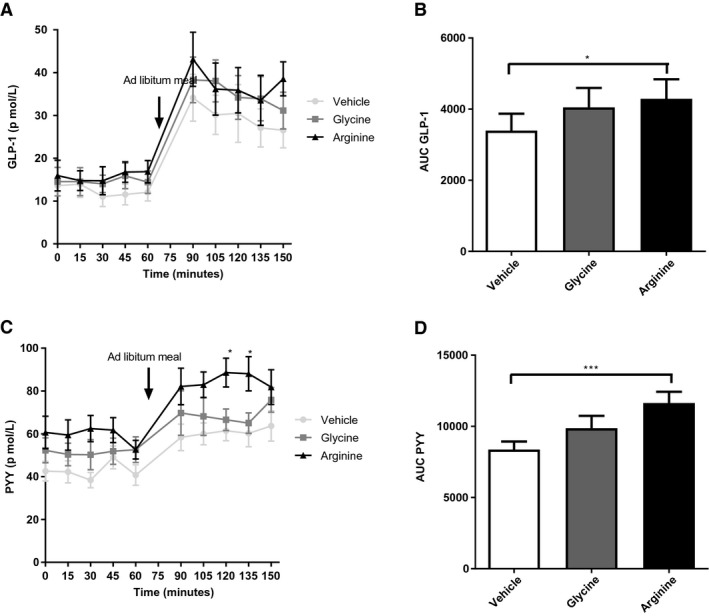
Effect of L‐arginine on gut hormone release in healthy volunteers following an *ad libitum *meal. Results of oral ingestion of vehicle, 17.1 mmol of arginine, or 17.1 mmol of glycine (*n *= 9) on (**A**) GLP‐1, (**B**) AUC of GLP‐1 (*P < 0.05), (**C**) PYY (*P < 0.05), and (**D**) AUC of PYY (***P < 0.001). Data are expressed as mean ± SEM.

L‐arginine significantly increased plasma GLP‐1 levels following ingestion of the *ad libitum* meal compared with levels following vehicle treatment (*P* < 0.05) (Figure 2A‐2B). L‐arginine significantly increased plasma PYY levels following administration of the *ad libitum* meal compared with levels following vehicle treatment (*P* < 0.001) (Figure 2C‐2D). While it is difficult to control for food intake in this analysis, AUC for change in GLP‐1 levels following the meal correlated with food intake with both vehicle control and L‐arginine ingestion (*R* = 0.71 and 0.75, respectively; *P* < 0.05 for both) but not following glycine ingestion (*P* = 0.88). There was no correlation between AUC for change in PYY levels and food intake with any treatment, suggesting that this effect was independent of food intake.

#### L‐arginine did not reduce acute food intake or affect subjective measures of appetite.

There was no difference in energy intake or subjective measures of appetite using VAS following ingestion of vehicle, 17.1 mmol of arginine, or 17.1 mmol of glycine (*n *= 9). Food consumption at the *ad libitum* meal was 929 ± 87.6 kcal (mean ± SEM) in the vehicle group compared with 1,154 ± 156.9 kcal in the glycine group and 992 ± 93.4 kcal in the L‐arginine group (*P* = 0.26).

#### Effects of L‐arginine on subsequent energy intake and fat intake.

**Figure 3:**
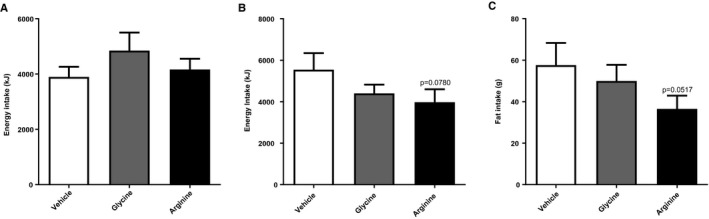
(**A**) Effect of L‐arginine on food intake at an *ad libitum *meal. (**B**) Twelve‐hour food intake following study visit (*P* = 0.0780; arginine vs. vehicle) and (**C**) twelve‐hour fat intake following study visit (*P *= 0.0517; arginine vs. vehicle) following ingestion of vehicle, 17.1 mmol of arginine, or 17.1 mmol of glycine (*n *= 9). Data are expressed as mean ± SEM.

Assessment of subsequent energy intake by self‐reported food diaries showed a trend toward lower energy intake and particularly fat intake (Figure 3), though these effects did not achieve statistical significance.

## Discussion

These data show that in combination with an *ad libitum* meal, L‐arginine significantly elevates GLP‐1 and PYY in healthy human volunteers compared with vehicle treatment. L‐arginine, at the doses administered, did not result in any serious side effects.

Appetite is regulated by complex central circuitry in response to several peripheral and central inputs. These include acute nutritional status, which is thought to be communicated to the appetite centers of the brain by signals such as changes in gut hormone release profile and vagal signaling as well as changes in circulating nutrients 36, 37. However, the precise stimuli for these signals and how they act in coordination and in the context of long‐term energy homeostasis are poorly understood. The effects observed in our study suggest L‐arginine may augment postprandial release of anorectic gut hormones. The mechanisms by which L‐arginine stimulates release of plasma GLP‐1 and PYY remain unclear 30. It is possible that L‐arginine is sensed by amino‐acid‐sensing receptors in the gut, which then mediate its effect on gut hormone release. Some of these receptors have been reported to be expressed on the same enteroendocrine cells that express GLP‐1 and PYY 38, 39, 40. These L cells are known to express a range of nutrient‐sensing receptors 19, and it may be that while L‐arginine alone does not have a significant effect on hormone release, it may potentiate the response to other nutrients ingested during the test meal. In addition, L‐arginine may possibly potentiate the effect of stomach distension on hormone release, which might also explain why differences are observed only postprandially. It is possible that the ability of L‐arginine to potentiate the effect of food intake on GLP‐1 release is reflected in the higher *R* value for the relationship between postprandial changes in GLP‐1 concentrations and food intake following L‐arginine ingestion, though further work would be required to confirm whether this is the case. It is interesting that there may be delayed effects of L‐arginine on appetite as suggested by the trends in the self‐reported food diaries. L‐arginine appears to potentially have effects on energy intake and fat intake subsequent to the study period. This may be a result of the postprandial effect on gut hormone release seen in the second study. Indeed, GLP‐1 analogues do suppress food intake and can do so for extended time intervals 41. L‐arginine has been shown to have a delayed and sustained anorectic effect in animals, with a reduction in food intake when food was returned 8 hours after administration in mice 31. Identifying food components that can increase postprandial GLP‐1 release may be useful in people with impaired glucose tolerance or type 2 diabetes, though further work is required to determine whether L‐arginine might have such utility, perhaps in combination with other nutrients.

A high‐protein meal consisting of 200 g of cooked chicken would be expected to contain 3 to 5 g of arginine, and hence doses of L‐arginine used (approximately 3 g) were comparable to those that might be ingested at a single high‐protein meal. However, we do acknowledge that the release profile of similar amounts of L‐arginine within the gut might be quite different from hypromellose capsules compared with that from protein ingestion. At these physiological doses, administration of L‐arginine in concert with other macronutrients (from the meal) results in the release of anorectic gut hormones. It is likely that the release of L‐arginine is a gradual process once it is in the small intestine, and it is difficult to know how far down the gastrointestinal tract significant concentrations of L‐arginine would reach before being absorbed. From our data, it is possible that L‐arginine is exerting its effect on gut hormone release at around 120 minutes; this may reflect the point at which it comes in direct contact with amino‐acid‐sensing L cells in the distal small intestine. There is a higher concentration of L cells in the distal small intestine 42, 43, and the elevation in PYY levels observed following a high‐protein meal is sustained for several hours 12, 15. It may be that this delayed effect is a result of direct sensing of amino acids in the distal small intestine, perhaps several hours after ingestion. L‐arginine is known to stimulate pancreatic hormone release when administered at higher levels to humans 44, 45, but the GLP‐1 detected should reflect the products of enteroendocrine cells rather than pancreatic alpha cells, as the assay used does not recognize glucagon or other preproglucagon products; however, it does detect all amidated forms of GLP‐1. The lack of suppression of ghrelin is in line with work carried out in our group looking at the effect of L‐arginine in rodents (unpublished data).

Further studies are required to investigate the effects of L‐arginine in combination with other macronutrients to determine whether this can result in a reduction in appetite. This study had a small number of participants as it was initially a pilot study to determine tolerability, with the second study being powered to detect a 30% change in gut hormone release. The majority of participants in these studies were women, and further work is required to determine whether similar effects occur in men. In addition, the volunteers will have had varied energy requirements, and thus there would be expected differences in their baseline food intake at an *ad libitum* meal; this is to some degree controlled for by the use of a crossover design, but it may be useful for future studies to measure the baseline energy expenditure of participants and to control for this in the food intake analysis. The dose of L‐arginine was chosen as it corresponded with levels found in a high‐protein meal and therefore allowed a physiological response to be observed. It is possible that higher doses of L‐arginine may have a greater effect on gut hormone release that would be sufficient to drive changes in appetite. However, 3 g of L‐arginine a day is also the maximum dose considered to be a foodstuff rather than a drug by the Medicines and Healthcare products Regulatory Agency, and effectiveness at this dose would facilitate the design of a supplement subject to food law rather than drug law. It is also possible that coadministering L‐arginine with other specific macronutrients may result in changes in gut hormone release sufficient to influence food consumption and satiety. The regulation of food intake is affected by a multitude of factors including the volume and composition of a meal, which can have synergistic effects on gut hormone release and appetite. While it seems that L‐arginine alone at the doses administered may not be useful in the suppression of appetite, further work is required to determine the effect of L‐arginine when combined with other nutrients on gut hormone release and appetite.

## Funding agencies:

This paper presents independent research funded by the Biotechnology and Biological Sciences Research Council (BBSRC), Medical Research Council (MRC), and Society for Endocrinology and supported by the National Institute for Health Research (NIHR) Clinical Research Facility (CRF) and Biomedical Research Centre (BRC) at Imperial College Healthcare National Health Service (NHS) Trust. AA is supported by an MRC Clinical Training Research Fellowship. The Section of Endocrinology and Investigative Medicine is funded by grants from MRC, BBSRC, NIHR, Integrative Mammalian Biology (IMB) Capacity Building Award, and an FP7‐ HEALTH‐ 2009‐ 241592 EuroCHIP grant, and it is supported by the NIHR BRC Funding Scheme. The views expressed are those of the author(s) and not necessarily those of the funders, NHS, NIHR, or the Department of Health.

## Disclosure:

The authors declared no conflict of interest.
